# Genetic diversity of *Microviridae* phages in the human respiratory tract

**DOI:** 10.3389/fcimb.2025.1629120

**Published:** 2025-09-08

**Authors:** Peiting Yang, Huanyan Zhang, Liang Yin, Jiaheng Chen, Yue Chen, Hongfeng Yang, Qi Liu, Wen Zhang

**Affiliations:** ^1^ Institute of Critical Care Medicine, The Affiliated People’s Hospital, Jiangsu University, Zhenjiang, China; ^2^ Department of Laboratory Medicine, School of Medicine, Jiangsu University, Zhenjiang, Jiangsu, China; ^3^ Department of Laboratory Medicine, Jintan Affiliated Hospital of Jiangsu University, Changzhou, Jiangsu, China; ^4^ Department of Breast Surgery, Affiliated People’s Hospital, Jiangsu University, Zhenjiang, China; ^5^ Department of Dermatology, The Affiliated Hospital of Jiangsu University, Zhenjiang, Jiangsu, China

**Keywords:** *Microviridae*, genome, major capsid protein, phylogenetic analysis, diversity, potential host bacteria

## Abstract

Recent studies have revealed that *Microviridae*, a family of ssDNA viruses, are widely distributed in natural environments and play significant roles in various ecosystems. While *Microviridae* members dominate the human gut microbiome, their genetic diversity in the human respiratory ecosystem remains unclear. The distribution, genetic characteristics, and ecological roles of *Microviridae* are still poorly understood. This study identified 327 Microviridae-associated contigs from nasopharyngeal swab samples of healthy individuals through metagenomic sequencing and comparative genomics analysis, including 15 near-complete *Microviridae*-related genomes. These genomes exhibited high sequence divergence from each other, revealing their high genetic diversity. Phylogenetic analysis based on VP1 (major capsid protein; F protein) demonstrated that the 15 genomes could be classified into seven distinct *Microviridae* groups. CRISPR spacer matching predicted the host of the 15 genomes. The total read counts of *Microviridae* across all 12 libraries were quantified and compared using the Kruskal-Wallis test. This work significantly expands the understanding of the diversity, genomic architecture, and evolutionary dynamics of *Microviridae* within the human respiratory tract.

## 1 Introduction

The family *Microviridae* comprises non-enveloped, round, T=1 icosahedral prokaryotic viruses (Doore and Fane, 2016) with circular single-stranded positive-sense DNA genomes. Based on morphology, biochemistry, biophysical properties, and genome size, *Microviridae* genomes are divided into two size ranges: Microvirus genomes are 5.3–6.1 kb, while gokushovirus genomes are considerably smaller, 4.4–4.9 kb. (ICTV *Microviridae*), with a total size range of approximately 4.4–6.1 kb. Replication occurs via dsDNA intermediates and rolling-circle mechanisms. The compact genomes of ssDNA viruses (up to 8.5 kb) enable shorter replication cycles (Zhong et al., 2015). These circular genomes typically encode fewer than 10 genes (Zhong et al., 2015), usually including functional proteins such as the major capsid protein (VP1) and replication-associated protein (VP4) (Labrie et al., 2014). The ICTV specifies that current species demarcation criteria rely on temperature and host range but acknowledges these may not be strictly applied (Prangishvili et al., 2017). Currently, the ICTV officially recognizes two subfamilies, Bullavirinae and Gokushovirinae. The subfamily Bullavirinae includes genera such as Alphatrevirus, Gequatrovirus, and Sinsheimervirus (Wang et al., 2019); the subfamily Gokushovirinae includes genera such as Bdellomicrovirus, Chlamydiamicrovirus, Enterogokushovirus and Spiromicrovirus. Although rare in environmental samples, Bullavirinae have been extensively studied, with the Gammaproteobacteria-infecting phage φX174 as a representative member which was found to build the capsids using proteins that fold as a “jelly roll” β-barrel (San Martín and van Raaij, 2018), the major capsid protein have the eight-stranded antiparallel β-sandwich motif (McKenna et al., 1994); In contrast, the Gokushovirinae subfamily is highly abundant in diverse environments and infects a broad range of hosts, including *Spiroplasma*, *Chlamydia*, *Bdellovibrio*, and Enterobacteriaceae (Olo Ndela et al., 2022). Small circular single-stranded DNA viruses of the *Microviridae* family are both prevalent and diverse in all ecosystems (Olo Ndela et al., 2022). The human respiratory tract represents a critical interface between the environment and host defense, where viral communities (virome) play pivotal roles in modulating immune responses and maintaining mucosal homeostasis (Shkoporov et al., 2019; Rastogi et al., 2022). Unlike the gut virome—which is dominated by bacteriophages like *Microviridae* and extensively characterized for its impact on metabolic and inflammatory diseases (Shkoporov et al., 2019)—the respiratory virome remains understudied despite its direct link to respiratory infections, asthma, and chronic obstructive pulmonary disease (Delwart, 2007). Respiratory viruses face unique ecological pressures, including rapid air exchange, variable temperature/humidity, and constant exposure to airborne pathogens, resulting in distinct community structures compared to the nutrient-rich, stable gut environment. Ecological studies have highlighted the dominance of microbial viruses in the biosphere, with their abundance and impact on microbial community composition and diversity earning them the title of “key players in global ecosystems” (Krupovic and Forterre, 2011). The human respiratory microbiota is closely linked to immune homeostasis and resistance against pathogen colonization (Rastogi et al., 2022). Metagenomic approaches to viral characterisation have been applied to respiratory secretions and have broadened the range of known viral diversity (Delwart, 2007). Through viral metagenomic analysis, we identified highly divergent *Microviridae* sequences in our samples. This study analyzes the genomic architecture, VP1-based sequence similarity (a classical taxonomic marker for *Microviridae*), and phylogenetic diversity of these phages, This study revealed the genomic evolutionary characteristics of *Microviridae* in the respiratory tract.

## 2 Materials and methods

### 2.1 Samples and sample treatment

To investigate the composition of respiratory viruses in healthy individuals, nasopharyngeal swab samples were collected from 140 healthy (All enrolled participants were asymptomatic, exhibiting no respiratory symptoms or comorbidities) subjects (aged 8–60 years) in Jiangsu Province, China, in 2024 (**Table 1**). The study protocol was approved by the Ethics Committee of Jiangsu University (Approval No. JSDX2023100702). Samples were collected by gently scraping the posterior pharyngeal wall and bilateral tonsils using sterile swabs, rotating 3–5 times to ensure adequate sampling. Swabs were stored in sterile collection tubes at 4°C. Prior to viral metagenomic analysis, swabs were immersed in 0.5 mL Dulbecco’s phosphate-buffered saline (DPBS), vortexed for 5 minutes, and incubated at 4°C for 30 minutes. After centrifugation at 15,000×ɡ for 10 minutes, supernatants were collected and stored at -80°C. For pooled analysis, 50 μL of supernatant from each of 14 samples was combined. The pooled sample was filtered through a 0.45 μm membrane, centrifuged (13,000×ɡ, 5 minutes), and 166.5 μL of virus-enriched fluid was collected. Filtrates were treated with a mixture of DNase, RNase, Benzonase, and Baseline-ZERO (37°C, 60 minutes) to digest unprotected nucleic acids (Zhang et al., 2014).

**Table 1:** We have summarized the demographic metadata of the sample libraries, including library ID, place of origin, age range, and geographic coordinates (longitude/latitude) of the sampling locations.

library ID	Place of origin of the Subjects	Age Range	Longitude and Latitude of the Sample Source Location
136J2	Jintan City, Jiangsu Province, China	aged 8–60 years	31.73 N, 119.52 E
146H2	Huai’an City, Jiangsu Province, China	aged 8–60 years	33.41 N, 118.90 E
144D2	Dongtaigang City, Jiangsu Province, China	aged 8–60 years	32.86 N, 120.31 E
140X3	Xuyi, Jiangsu Province, China	aged 8–60 years	32.97 N, 118.54 E
139X2	Xuyi, Jiangsu Province, China	aged 8–60 years	32.97 N, 118.54 E
149B2	Baoying City, Jiangsu Province, China	aged 8–60 years	33.24 N, 119.42 E
142Z2	Zhangjiagang City, Jiangsu Province, China	aged 8–60 years	31.88 N, 120.62 E
134D3	Donghai City, Jiangsu Province, China	aged 8–60 years	34.46 N, 118.78 E
133D2	Donghai City, Jiangsu Province, China	aged 8–60 years	34.46 N, 118.78 E
153L3	Liyang City, Jiangsu Province, China	aged 8–60 years	31.42 N, 119.37 E

### 2.2 Library construction and bioinformatic analysis

Total nucleic acids were extracted using the QIAamp MinElute Viral Kit (Qiagen). Libraries were prepared with the Nextera XT DNA Library Preparation Kit (Illumina) and sequenced on an Illumina NovaSeq platform with 150 bp paired-end reads.

**Figure 1:**
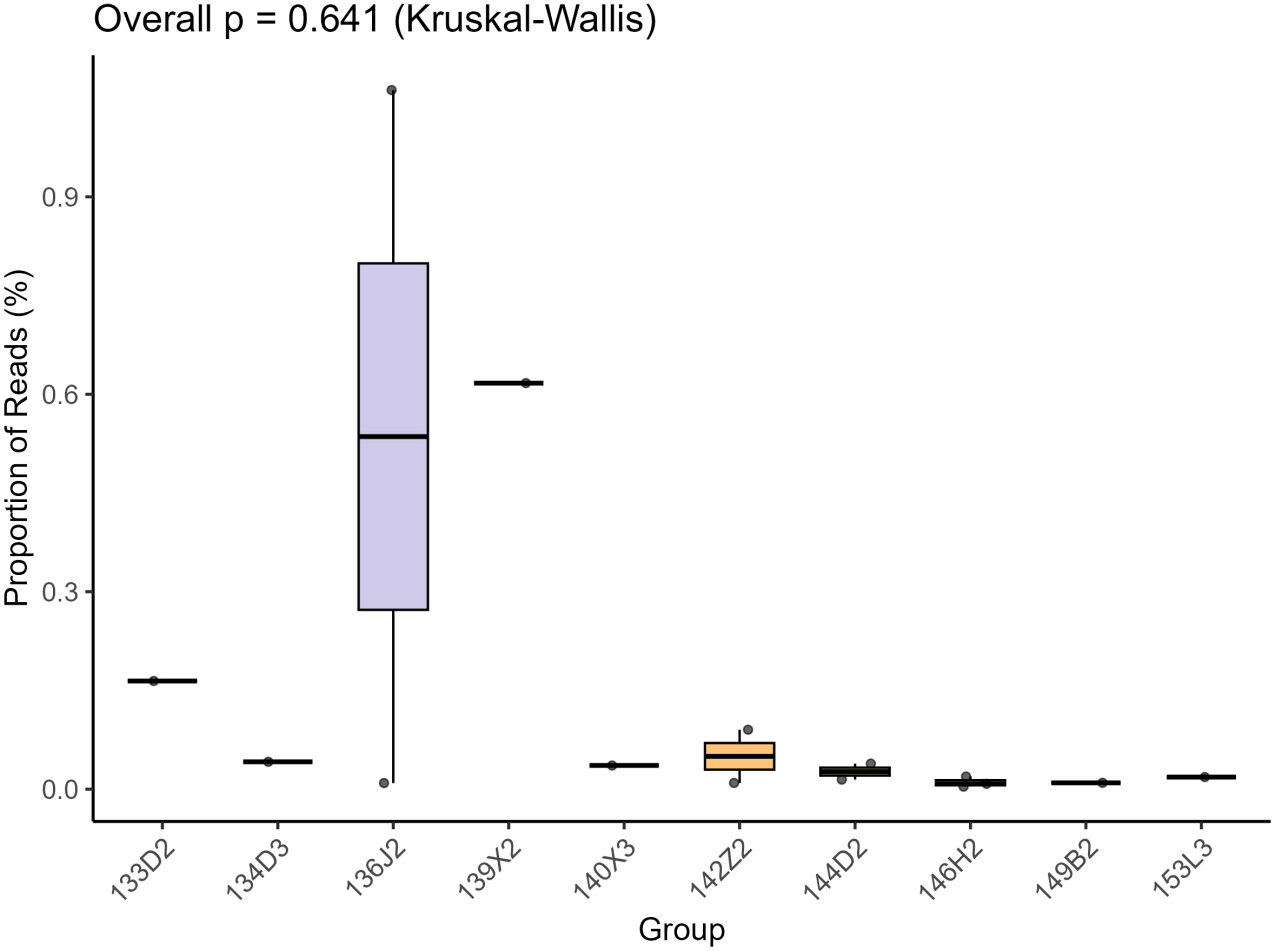
This is a box-plot based on the Kruskal - Wallis test, which is used to show the distribution of the proportion of Reads (%) among different groups (Group, including groups such as 135D2, 13423, etc.). The vertical axis represents the proportion of Reads, and the horizontal axis represents the groups. The overall test p - value (Overall p) is 0.641. The box - plot presents the distribution characteristics of the median, quartiles, and outliers of the data in each group. It can be used to intuitively compare the differences and degree of dispersion of the proportion of Reads among different groups.

For bioinformatic analysis, raw sequencing reads were processed using quality parameters where 93.2% of reads exceeded Q30. Raw data were demultiplexed using Illumina’s official software, followed by removal of clonal reads and low-quality ends (Phred quality score threshold = 10). Adapter sequences were trimmed using VecScreen (NCBI BLASTn-specific parameters). Cleaned reads were *de novo* assembled using the ENSEMBLE assembler. Contigs and unassembled reads were aligned to a custom viral proteome database (integrating NCBI viral reference proteomes and non-redundant viral sequences) via BLASTx (E-value <10^-5^). Following the acquisition of continuous sequences, assembly was performed using the most stringent parameters, thus sequence mismatches were strictly prohibited. Any ambiguous bases identified in the assembled sequences were immediately validated via conventional PCR coupled with Sanger sequencing. This protocol ensures the exclusion of chimeric sequences arising from sample cross-contamination. Although individual libraries generated more than two genomic sequences, these sequences lack nucleotide-level homology, thereby preventing misassembly errors. All 12 libraries tested positive for *Microviridae*. The total read counts of *Microviridae* across all 12 libraries were quantified and compared using the Kruskal-Wallis test. Neither the *post-hoc* pairwise comparisons nor the overall p-value demonstrated statistical significance (**Figure 1**). Candidate viral sequences were further compared against a non-viral protein database (NVNR) to exclude false positives, yielding high-confidence sequences. Sequences with significant hits to *Microviridae* were validated for circular genome characteristics (overlapping terminal reads) in Geneious 2025.0.2. Non-circular contigs >3,000 bp were extended by mapping raw reads and manually verified for circularity. Sequences >3,500 bp encoding the major capsid protein VP1 were classified as target genomes.

### 2.3 Phylogenetic analysis

In the phylogenetic analysis, relevant protein sequences were aligned using the NCBI BLASTx tool against the GenBank database. The best matches were selected as reference sequences. The reference sequences selected for phylogenetic tree construction all belong to the family *Microviridae*, requiring complete VP1 gene annotations with RefSeq-curated status and representing distinct ICTV-recognized taxa. And major capsid protein sequences of reference strains from different genera within the family *Microviridae* were downloaded from the NCBI GenBank database. Alignments were refined using MUSCLE in MEGA-X (Kumar et al., 2018), with gapped positions temporarily excluded. A Bayesian inference tree was constructed using MrBayes v3.2.7 with specified parameters.

## 3 Results

**Figure 2:**
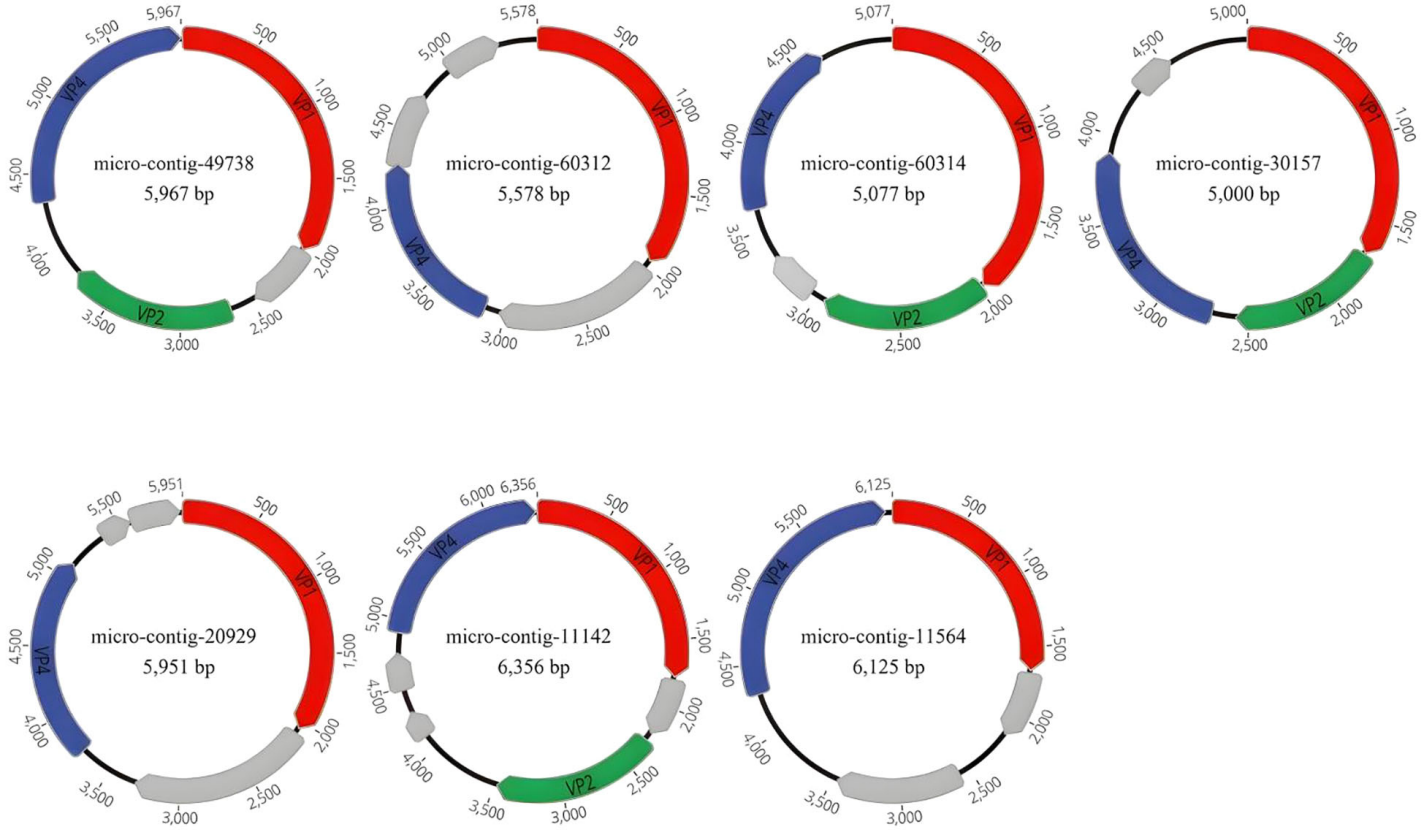
In this study, the genomic architectures of 15 phages were identified, with seven representative sequences selected. Proteins encoded by the viruses were annotated, and those lacking homology to proteins in the NCBI database were designated as hypothetical proteins. VP1 is labeled in red, VP2 in green, VP4 in blue, and the hypothetical protein is marked in gray. The uncharacterized grey ORFs are all hypothetical proteins. Arrows indicate the coding direction of genes.

We assembled 15 complete genomes (Genome circularity was confirmed using Geneious, with repeats exceeding 18 bp identified at the termini of the assembled sequences, suggesting circular genome topology. For conserved ORFs, all 15 genomic sequences contained VP1 and VP4, while 8 additionally harbored VP2. Their presence was validated via NCBI BLAST alignments. Collectively, circularity and conserved ORFs validate genome integrity.) of *Microviridae* from 12 libraries using the low sensitivity/fastest parameters in Geneious 2025.0.2, which have been deposited in NCBI under accession numbers PV594030 to PV594044. The 15 genomes ranged in length from 5,000 nt to 6,484 nt, with GC contents ranging from 33.5% to 47.6% (**Table 2**). We annotated the gene structures of seven representative sequences from seven distinct groups, each encoding 4 to 6 open reading frames (ORFs). (**Figure 2**). All 15 representative sequences contained the major capsid protein (VP1, designated as F protein), along with DNA pilot protein (VP2, H protein), replication-associated protein, replication initiation protein and replication protein (VP4, A protein) auxiliary genes. The conserved VP1 protein in *Microviridae* serves as a target for identifying these phages in submitted metagenomic databases.

**Table 2:** Summary table of the length, CG content, protein-coding sequences (excluding hypothetical proteins), and the best matches obtained after NCBI BLASTX for 15 complete *Microviridae* genomes.

Genome ID	Genome length	GC content	Protein-coding sequence	Best match
micro-contig-49728	5,116 nt	36.8%	VP1-VP2-VP4	UYL88497
micro-contig-49738	5,967 nt	47.6%	VP1-VP2-VP4	DAF24915
micro-contig-57501	5,685 nt	37.1%	VP1-VP2-VP4	WP369397756
micro-contig-60312	5,578 nt	37.6%	VP1-VP4	WP369397756
micro-contig-60314	5,077 nt	38.1%	VP1-VP2-VP4	WP297123521
micro-contig-40521	6,124 nt	33.5%	VP1-VP4	DAN45594
micro-contig-52438	5,647 nt	36.2%	VP1-VP4	DAI49539
micro-contig-30157	5,000 nt	34.4%	VP1-VP2-VP4	WMC01482
micro-contig-20443	5,909 nt	36.6%	VP1-VP4	DAM86282
micro-contig-20929	5,951 nt	38.7%	VP1-VP4	WMC01578
micro-contig-7045	6,484 nt	46.5%	VP1-VP2-VP4	DAS04622
micro-contig-59793	6,043 nt	36.9%	VP1-VP2-VP4	DAS05872
micro-contig-11142	6,356 nt	36.4%	VP1-VP2-VP4	DAK17497
micro-contig-11564	6,125 nt	33.9%	VP1-VP4	WP278485743
micro-contig-13304	5,885 nt	35.8%	VP1-VP4	DAJ34221

The seven representative sequences from the seven groups are highlighted in red font.

**Figure 3:**
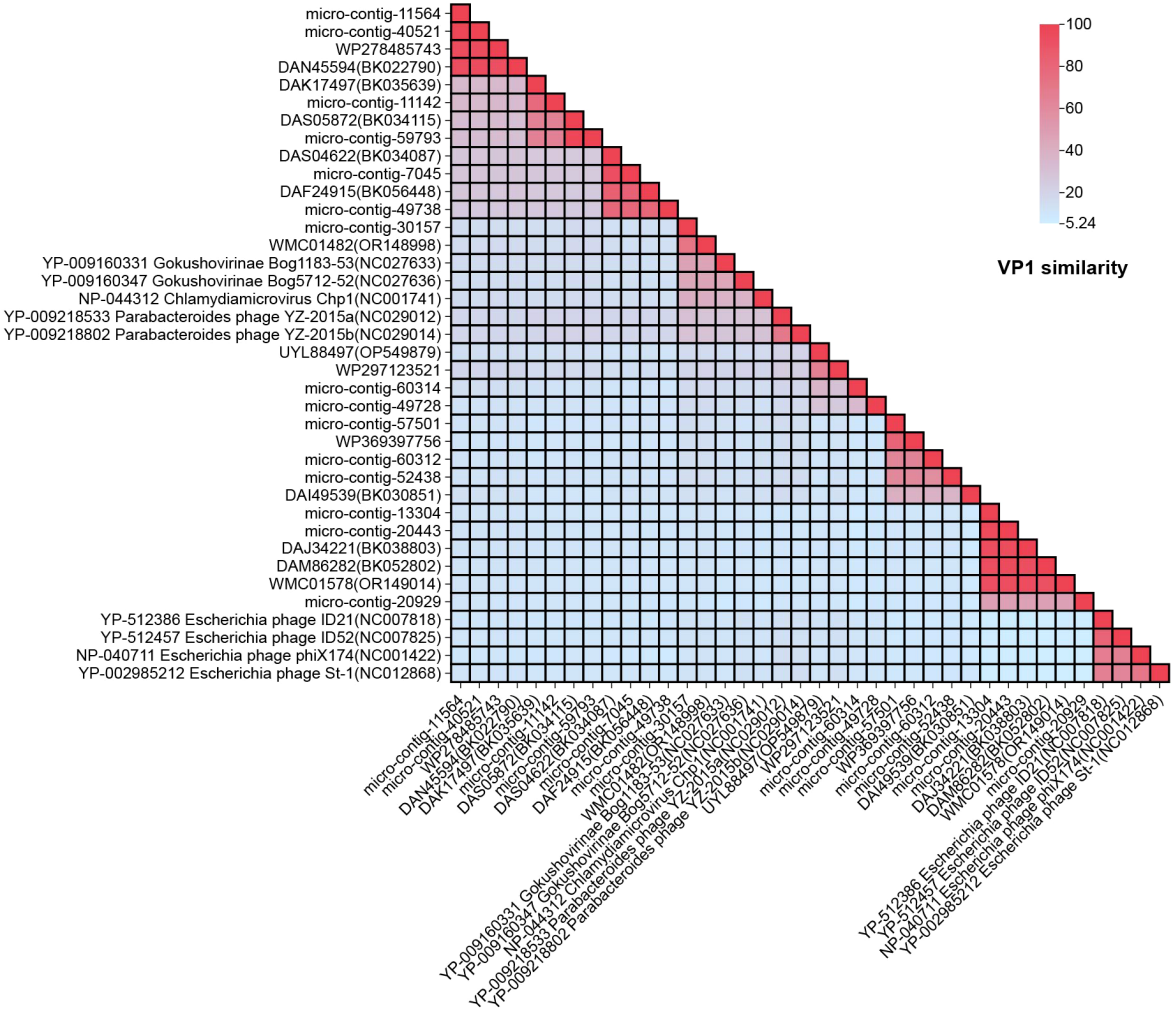
Pairwise similarity comparison of the 15 phage genomes identified in this study: Amino acid genomes of the 15 phages were compared against nine reference strains from different *Microviridae* genera and 14 homologous sequences. Sequence alignment was performed using NCBI’s BLASTx tool. “Percent Identity” and “Divergence” were calculated with default parameters, and results were visualized using Chiplot.

To determine the relationship between the 15 genomes and members of the family *Microviridae*, amino acid sequences of VP1 proteins were aligned and analyzed (**Figure 3**). The VP1 protein similarity and whole-genome similarity between our strains and their closest relatives ranged from 41.05%-97.79% (BLASTx) and 74.83%-95.99% (BLASTn), The results showed that the highest amino acid sequence similarity between VP1 proteins of all phages and reference strains from different *Microviridae* genera was below 46.73%. Based on the amino acid sequence of VP1, for example, micro-contig-30157 exhibited the highest amino acid sequence identity of 46.73% with Gokushovirinae Bog1183 - 53 (NC_027633) when its VP1 was compared. Similarly, micro-contig-11142 showed a highest identity of 19.85% when its VP1 was aligned with Parabacteroides phage YZ-2015b (NC_029014), while micro-contig-20929 only had 10.73% identity when its VP1 was compared to the same reference strain.

**Figure 4:**
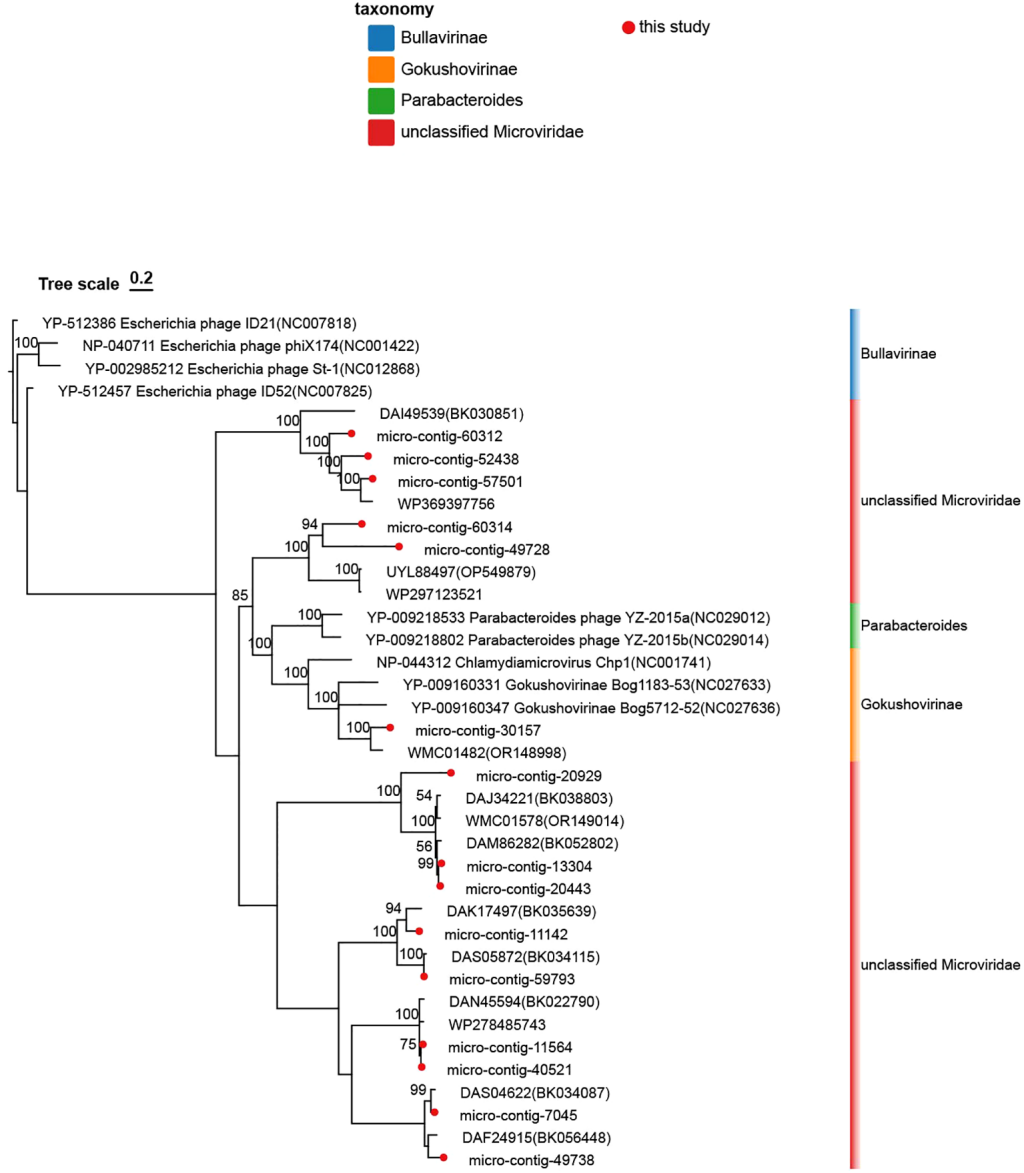
Phylogenetic analysis of the 15 *Microviridae* phages identified in this study. This tree was constructed based on the amino acid sequences of VP1 from the identified phages and reference strains of the family *Microviridae*. The *Microviridae* phages identified in this study are marked with red dots. Branches representing the subfamily Bullavirinae are denoted by blue blocks, Gokushovirinae by orange blocks, Parabacteroides by green blocks, and unclassified Microviridae by red blocks. Taxonomic labels at the genus level and subfamily separation are explicitly annotated. The scale bar indicates nucleotide substitutions per site. Branch support values (bootstrap/posterior probabilities) are displayed for all nodes.

The VP1-based phylogenetic tree (including nine reference strains) revealed four strains belonging to Bullavirinae (rooted with any of these four), two strains to Parabacteroides, and three strains to Gokushovirinae. Reference strains from various *Microviridae* genera are included (**Figure 4**). Phylogenetic analysis revealed that the 15 genomes formed seven distinct clades, which were clustered into seven groups. Specifically: micro-contig-60312 clustered closely with strain DAI49539 (BK030851), micro-contig-60314 with strain UYL88497 (OP549879), micro-contig-30157 with Gokushovirinae strain NC027636, micro-contig-20929 with strain DAJ34221 (BK038803), micro-contig-11142 with strain DAK17497 (BK035639), micro-contig-11564 with strain DAN45594 (BK022790), micro-contig-49738 with strain DAF24915 (BK056448).

## 4 Discussion

**Figure 5:**
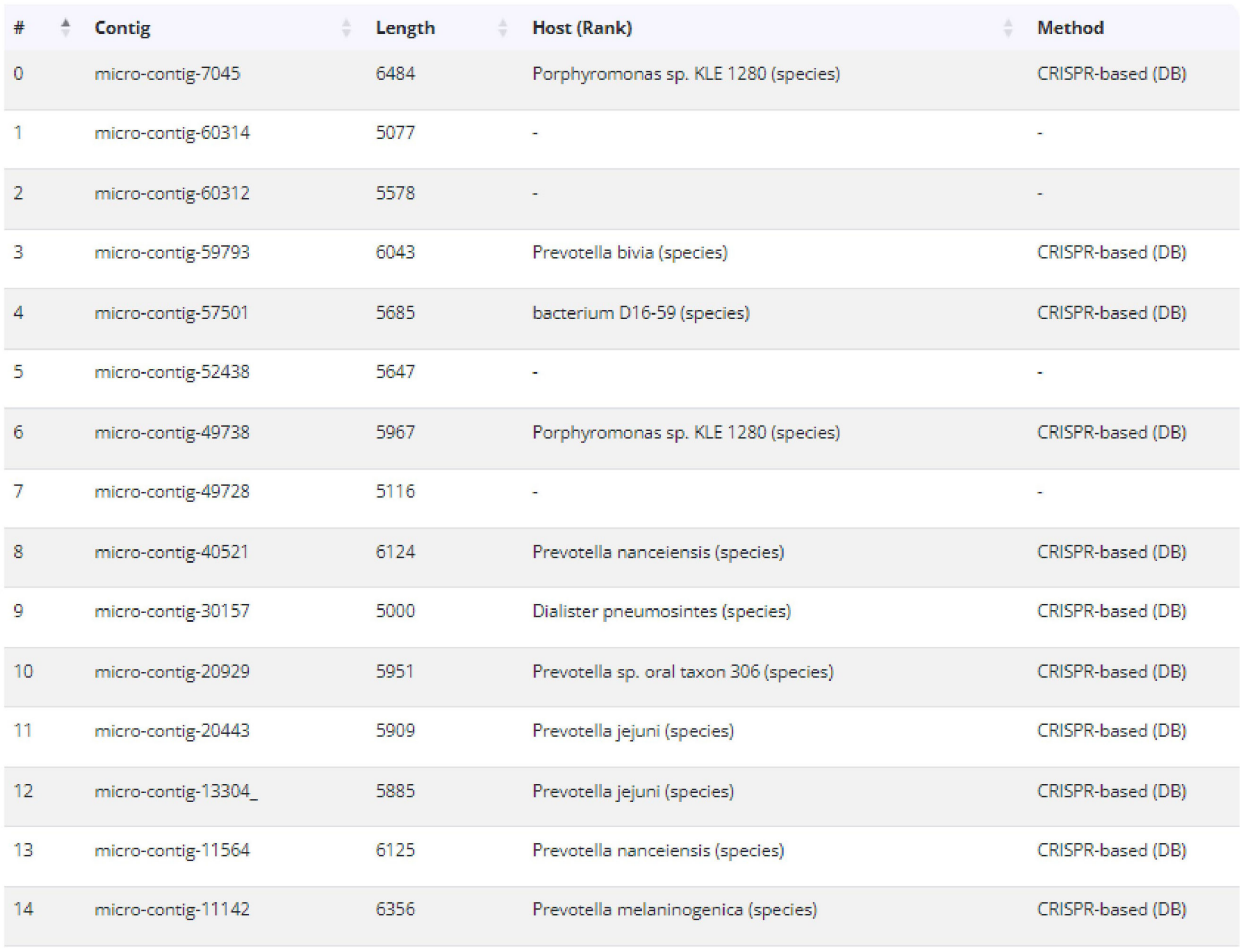
Host prediction result via CRISPR spacer matches was further investigated and refined. Potential hosts include: Porphyromonas sp. KLE 1280 (species), Prevotella bivia (species), bacterium D16 - 59 (species), Porphyromonas sp. KLE 1280 (species), Prevotella nanceiensis (species), Dialister pneumosites (species), Prevotella sp. oral taxon 306 (species), Prevotella jejuni (species), Prevotella melaninogenica (species).

In summary, we detected and extracted 15 phage sequences from the nasopharyngeal swab samples of healthy humans, and characterized their complete genomes, shifting the research on microviruses from the intestinal system to the respiratory system. Previous research on phages has provided a new potential antimicrobial candidate for pathogenic Vibrio parahaemolyticus, and has provided a theoretical basis for intervention strategies for digestive tract diseases such as acute gastroenteritis based on *Microviridae* phages. Similarly, it can also provide a theoretical basis for the development of intervention strategies for respiratory diseases based on *Microviridae* phages (Shkoporov et al., 2019). Current knowledge indicates that *Prevotella* and *Bacteroides* can serve as hosts for both crAss-like phages and *Microviridae* (Shkoporov et al., 2019), *Escherichia coli (* Labrie et al., 2014), pathogenic *Vibrio parahaemolyticus (* Guo et al., 2022), *Shigella flexneri (* Lu et al., 2022), *Bdellovibrio bacteriovorus (* Brentlinger et al., 2002) can also serve as hosts for *microviridae*. Host prediction via CRISPR spacer matches was further investigated and refined. As depicted in (**Figure 5**), *Prevotella* was confirmed as a predicted host. In recent years, metagenomic sequence analysis has become a critical tool for studying viral diversity, including bacteriophages and archaeal viruses (Rokyta et al., 2006). Previous studies suggest that the observed similarities in the structures of viral capsid proteins may provide a basis for the natural classification of viruses. As a result, they have put forward the hypothesis that the structures of viral capsid proteins independently support the definition of the natural classification of viruses (Sinclair et al., 2017). Peter Simmonds and colleagues proposed that future ICTV classification frameworks should incorporate metagenomics-derived taxonomy based on genomic features (Iranzo et al., 2016; Aiewsakun et al., 2018). In recent years, classification systems based on genomic architecture have increasingly replaced traditional morphological criteria, offering robust guidance for viruses with highly divergent genomic sequences and organizations. All 15 phage sequences encode the VP1 protein, which is conserved among *Microviridae* members and may play a critical role in enhancing the pathogenicity of lysogenic bacteria and the global persistence of Gokushovirinae (Kirchberger and Ochman, 2020). *Microviridae* are likely opportunistic viruses (lysogenic or pseudo-lysogenic, capable of transitioning from rare/undetectable to dominant under favorable conditions), indicating their pivotal role in host mortality and community structure (Zhong et al., 2015). The genomic architecture of respiratory *Microviridae* exhibits remarkable plasticity, characterized by compact genomes (5,000–6,356 nt) encoding 4–6 open reading frames. Homology comparison based on the VP1 protein revealed that the maximum amino acid sequence similarity between these 15 phages and other reference strains within the *Microviridae* family, as well as among the phages themselves, was below 46.73%. Phylogenetic analysis of VP1, a conserved taxonomic marker, further supports the genetic distinctiveness of respiratory *Microviridae*. Such divergence challenges existing classification frameworks traditionally reliant on host range and morphological criteria, emphasizing the necessity of genomic-based taxonomy to accommodate novel lineages. Phylogenetic analysis demonstrated distant genetic relationships between the 15 phages identified in this study and reference strains from other *Microviridae* genera. Respiratory tract-associated microviruses are genetically distinct from their gastrointestinal counterparts. Based on genetic distance criteria, these phages may belong to novel genera within the *Microviridae* family.

Future studies should prioritize expanding the genomic database of respiratory *Microviridae* to refine phylogenetic boundaries and elucidate mechanisms underlying their genomic plasticity. Integrating structural analyses of capsid proteins or replication-associated proteins could further resolve taxonomic ambiguities. This demonstrates that research on *Microviridae* represents a significant advance in innovation. The detection of these phages in the respiratory tract suggests: *Microviridae* are not confined to the human intestinal tract but also colonize the respiratory system. This may stimulate experts and scholars to explore broader ecological niches, uncover novel possibilities, and contribute substantially to microbiology. Future interventions for respiratory diseases could target phage therapy, providing a theoretical foundation for developing *Microviridae*-based strategies. This approach may also inspire innovative drug development paradigms. Our study establishes the foundation for investigating the ecological and evolutionary roles of *Microviridae* in respiratory health. Ultimately, this study establishes a foundation for exploring the ecological and evolutionary roles of *Microviridae* in respiratory health and demonstrates the applicability of viral metagenomic approaches for phage discovery and characterization.

## Data Availability

The datasets presented in this study can be found in online repositories. The names of the repository/repositories and accession number(s) can be found in the article/supplementary material.

## References

[B1] Aiewsakun P. Adriaenssens E. M. Lavigne R. Kropinski A. M. Simmonds P. (2018). Evaluation of the genomic diversity of viruses infecting bacteria, archaea and eukaryotes using a common bioinformatic platform: steps towards a unifed taxonomy. J. Gen. Virol. 99, 1331–1343. doi: 10.1099/jgv.0.001110, PMID: 30016225 PMC6230767

[B2] Brentlinger K. L. Hafenstein S. Novak C. R. Fane B. A. Borgon R. McKenna R.. (2002). *Microviridae*, a family divided: isolation, characterization, and genome sequence of phiMH2K, a bacteriophage of the obligate intracellular parasitic bacterium Bdellovibrio bacteriovorus. J. Bacteriol. 184, 1089–1094. doi: 10.1128/jb.184.4.1089-1094.2002, PMID: 11807069 PMC134817

[B3] Delwart E. L. (2007). Viral metagenomics. Rev. Med. Virol. 17, 115–131. doi: 10.1002/rmv.532, PMID: 17295196 PMC7169062

[B4] Doore S. M. Fane B. A. (2016). The *microviridae*: Diversity, assembly, and experimental evolution. Virology 491, 45–55. doi: 10.1016/j.virol.2016.01.020, PMID: 26874016

[B5] Guo R. Zheng K. Luo L. Liu Y. Shao H. Guo C.. (2022). Characterization and Genomic Analysis of ssDNA Vibriophage vB_VpaM_PG19 within *Microviridae*, Representing a Novel Viral Genus. Microbiol. Spectr. 10, e0058522. doi: 10.1128/spectrum.00585-22, PMID: 35862991 PMC9431446

[B6] Iranzo J. Koonin E. V. Prangishvili D. Krupovic M. (2016). Bipartite network analysis of the archaeal virosphere: evolutionary connections between viruses and capsidless mobile elements. J. Virol. 90, 11043–11055. doi: 10.1128/JVI.01622-16, PMID: 27681128 PMC5126363

[B7] Kirchberger P. C. Ochman H. (2020). Resurrection of a global, metagenomically defined gokushovirus. Elife 9, e51599. doi: 10.7554/eLife.51599, PMID: 32101162 PMC7062461

[B8] Krupovic M. Forterre P. (2011). *Microviridae* goes temperate: microvirus-related proviruses reside in the genomes of Bacteroidetes. PloS One 6, e19893. doi: 10.1371/journal.pone.0019893, PMID: 21572966 PMC3091885

[B9] Kumar S. Stecher G. Li M. Knyaz C. Tamura K. (2018). Mega X: molecular evolutionary genetics analysis across computing platforms. Mol. Biol. Evol. 35, 1547. doi: 10.1093/MOLBEV/MSY096, PMID: 29722887 PMC5967553

[B10] Labrie S. J. Dupuis MÈ Tremblay D. M. Plante P. L. Corbeil J. Moineau S. (2014). A new *Microviridae* phage isolated from a failed biotechnological process driven by Escherichia coli. Appl. Environ. Microbiol. 80, 6992–7000. doi: 10.1128/AEM.01365-14, PMID: 25192988 PMC4249002

[B11] Lu H. Xiong W. Li Z. Yan P. Liu R. Liu X. (2022). Isolation and characterization of SGF3, a novel *Microviridae* phage infecting Shigella flexneri. Mol. Genet. Genomics 297, 935–945. doi: 10.1007/s00438-022-01883-5, PMID: 35522301

[B12] McKenna R. Ilag L. L. Rossmann M. G. (1994). Analysis of the single-stranded DNA bacteriophage φX174, refined at a resolution of 3.0 Å. J. Mol. Biol. 237, 517–543. doi: 10.1006/jmbi.1994.1253, PMID: 8158636

[B13] Olo Ndela E. Roux S. Henke C. Sczyrba A. Sime Ngando T. Varsani A.. (2022). Reekeekee- and roodoodooviruses, two different Microviridae clades constituted by the smallest DNA phages. Virus Evol. 9, veac123. doi: 10.1093/ve/veac123, PMID: 36694818 PMC9865509

[B14] Prangishvili D. Bamford D. H. Forterre P. Iranzo J. Koonin E. V. Krupovic M. (2017). The enigmatic archaeal virosphere. Nat. Rev. Microbiol. 15, 724–739. doi: 10.1038/nrmicro.2017.125, PMID: 29123227

[B15] Rastogi S. Mohanty S. Sharma S. Tripathi P. (2022). Possible role of gut microbes and host’s immune response in gut-lung homeostasis. Front. Immunol. 13. doi: 10.3389/fimmu.2022.954339, PMID: 36275735 PMC9581402

[B16] Rokyta D. R. Burch C. L. Caudle S. B. Wichman H. A. (2006). Horizontal gene transfer and the evolution of microvirid coliphage genomes. J. Bacteriol. 188, 1134–1142. doi: 10.1128/JB.188.3.1134-1142.2006, PMID: 16428417 PMC1347346

[B17] San Martín C. van Raaij M. J. (2018). The so far farthest reaches of the double jelly roll capsid protein fold. Virol. J. 15, 181. doi: 10.1186/s12985-018-1097-1, PMID: 30470230 PMC6260650

[B18] Shkoporov A. N. Clooney A. G. Sutton T. D. S. Ryan F. J. Daly K. M. Nolan J. A.. (2019). The human gut virome is highly diverse, stable, and individual specific. Cell Host Microbe 26, 527–541.e5. doi: 10.1016/j.chom.2019.09.009, PMID: 31600503

[B19] Sinclair R. M. Ravantti J. J. Bamford D. H. (2017). Nucleic and amino acid sequences support structure-based viral classification. J. Virol. 91, e02275–e02216. doi: 10.1128/JVI.02275-16, PMID: 28122979 PMC5375668

[B20] Wang H. Ling Y. Shan T. Yang S. Xu H. Deng X.. (2019). Gut virome of mammals and birds reveals high genetic diversity of the family *Microviridae* . Virus Evol. 5, vez013. doi: 10.1093/ve/vez013, PMID: 31191981 PMC6555873

[B21] Zhang W. Li L. Deng X. Kapusinszky B. Pesavento P. A. Delwart E. (2014). Faecal virome of cats in an animal shelter. J. Gen. Virol. 95, 2553–2564. doi: 10.1099/vir.0.069674-0, PMID: 25078300 PMC4202271

[B22] Zhong X. Guidoni B. Jacas L. Jacquet S. (2015). Structure and diversity of ssDNA *Microviridae* viruses in two peri-alpine lakes (Annecy and Bourget, France). Res. Microbiol. 166, 644–654. doi: 10.1016/j.resmic.2015.07.003, PMID: 26226335

